# Functional Diversity and Microbial Activity of Forest Soils that Are Heavily Contaminated by Lead and Zinc

**DOI:** 10.1007/s11270-016-3051-4

**Published:** 2016-08-29

**Authors:** Marek Pająk, Ewa Błońska, Magdalena Frąc, Karolina Oszust

**Affiliations:** 1 Department of Forest Ecology and Reclamation, University of Agriculture in Krakow, Al. 29-go Listopada 46, 31-425 Kraków, Poland; 2 Department of Forest Soil, Faculty of Forestry, University of Agriculture in Krakow, Al. 29-go Listopada 46, 31-425 Kraków, Poland; 3 Institute of Agrophysics Polish Academy of Sciences, Doświadczalna 4, 20-290 Lublin, Poland

**Keywords:** Soil contamination, Dehydrogenase and urease activities, Microbial functional diversity

## Abstract

The objective of this study was to assess the impact of metal contamination on microbial functional diversity and enzyme activity in forest soils. This study involved the evaluation of the influence of the texture, carbon content and distance to the source of contamination on the change in soil microbial activity, which did not investigate in previous studies. The study area is located in southern Poland near the city of Olkusz around the flotation sedimentation pond of lead and zinc at the Mining and Metallurgical Company “ZGH Bolesław, Inc.”. The central point of the study area was selected as the middle part of the sedimentation pond. The experiment was conducted over a regular 500 × 500-m grid, where 33 sampling points were established. Contents of organic carbon and trace elements (Zn, Pb and Cd), pH and soil texture were investigated. The study included the determination of dehydrogenase and urease activities and microbial functional diversity evaluation based on the community-level physiological profiling approach by Biolog EcoPlate. The greatest reduction in the dehydrogenase and urease activities was observed in light sandy soils with Zn content >220 mg · kg^−1^ and a Pb content > 100 mg · kg^−1^. Soils with a higher concentration of fine fraction, despite having the greatest concentrations of metals, were characterized by high rates of Biolog®-derived parameters and a lower reduction of enzyme activity.

## Introduction

High heavy metal contamination negatively affects the circulation of nutrients, which reduces the amount of soil microorganisms, potentially changing their metabolic activity (Bååth, 1989; Kandeler et al., 1996; Wyszkowska and Wyszkowski, 2003; Guo et al., 2012). The studies of Kuperman and Carreiro (1997), Šmejkalová et al. (2003) and Sardar et al. (2007) showed that microbial activity was inhibited by heavy metals, such as Zn, Cd and Pb. According to Sardar et al. (2010), high concentrations of heavy metals (Cd and Pb) change soil microbial community structure and activities. The soil enzymatic activities decreased significantly with the increasing contamination of heavy metals (Pb, Zn, Ag, Cu and Cd), especially dehydrogenase and urease activities (Chen et al., 2005). Microbial parameters may be affected not only by heavy metals but also by soil properties, such as pH and carbonates, organic carbon and total nitrogen content (Moreno et al., 2001; Chodak et al., 2013). Renella et al. (2003) reported that the inhibition of enzyme activity by heavy metals was greater in sandy rather than in finer textured soils. Soil organic matter is the most important parameter controlling heavy metal behaviour in soils. Heavy metals bound on insoluble humic substances are relatively immobile (Borůvka and Drábek, 2004). The most important factor which affects heavy metals mobility is clay content. Clay minerals play a role as carriers of associated oxides and humic substances forming organo-mineral complexes which present different sorption capacities (Violante et al., 2010).

Soil enzymatic activities are sensitive bio-indicators of any natural and anthropogenic disturbance (Hinojosa et al., 2004; Kumar et al., 2013). Soil enzymes are important for catalysing several vital reactions that are necessary for microorganisms in soils as well as the stabilization of soil structure, organic waste decomposition, organic matter formation and nutrient cycling (Dick et al., 1996; Dick, 1997; Siczek and Frąc, 2012). Wolińska et al. (2015), Wolińska and Stępniewska (2012) and Veres et al. (2013) showed that soil enzyme activities are “sensors” of soil organic matter (SOM) decomposition. Decomposition of organic matter in soil depends on substance properties and accessibility of microorganisms and their enzymes. According to Brookes (1995), dehydrogenase activity can be used as an indicator of heavy metal contamination of soil. The results of Shen et al. (2005) indicated that urease activity may be an effective preliminary mechanism to estimate Cd, Zn, Pb and PAH pollution in the soil of zinc mines. The inhibition of urease is especially strong in the presence of certain heavy metals due to the changes in the molecular structure of the enzyme caused by the inhibitors. Metal pollution has been shown to affect microbial functional diversity (Stefanowicz et al., 2008). These authors found that bacterial functional diversity significantly decreased, whereas fungal functional diversity slightly increased with increasing metal concentrations.

The study area is the area with the heaviest contamination of Zn and Pb in Poland. Previous studies did not investigate the effect of texture variability, carbon content and distance from the contamination source on the microbial activity in forest ecosystems. No information is available regarding the content of Zn and Pb, which clearly inhibit microbial activity (Niklińska et al. 2006; Stefanowicz et al. 2008).

The objective of this study was to assess the impact of metal contamination on the microbial functional diversity and enzyme activity in forest soils. This study involved evaluation of the influence of the texture, carbon content and distance to the source of contamination on the change in soil microbial activity. The following hypotheses were tested in this paper: (1) an increase in the clay and organic carbon content in soil causes a less negative impact of Zn and Pb on microbial activity compared to that in soil with high sand content; (2) decreasing distance from the source of contamination caused a decrease in the soil microbial activity.

## Material and Methods

### Soil Sampling Sites

The study area is located in southern Poland near the city of Olkusz (N50° 17′ 3.67″; E19° 29′ 47.43″) and characterized by the following conditions of climate: the average precipitation in the months (IV–X) amounts to 515 mm, the average temperature is 12.5 °C. This area includes forests around a flotation sedimentation pond of lead and zinc at the Mining and Metallurgical Company “ZGH Bolesław, Inc.”. The pine forest was predominant in the study area (the pine was 20–50 years old). The central point of the study area was selected as the middle part of the sedimentation pond. The sampling was designed over a regular 500 × 500-m grid, where 33 sampling points were designated. The samples for laboratory analysis were collected in August 2014.

For each sampling point, the coordinates (*x*, *y*) were determined. Soil samples from the depth of 0–15 cm were taken below the organic horizon from the sampling points. From each sampling point, five subsamples were taken to form a composite sample. In addition, the distances from each sampling point to the sedimentation pond (Dist. 1) and the steelworks of ZGH Boleslaw (Dist. 2) were specified.

**Fig. 1:**
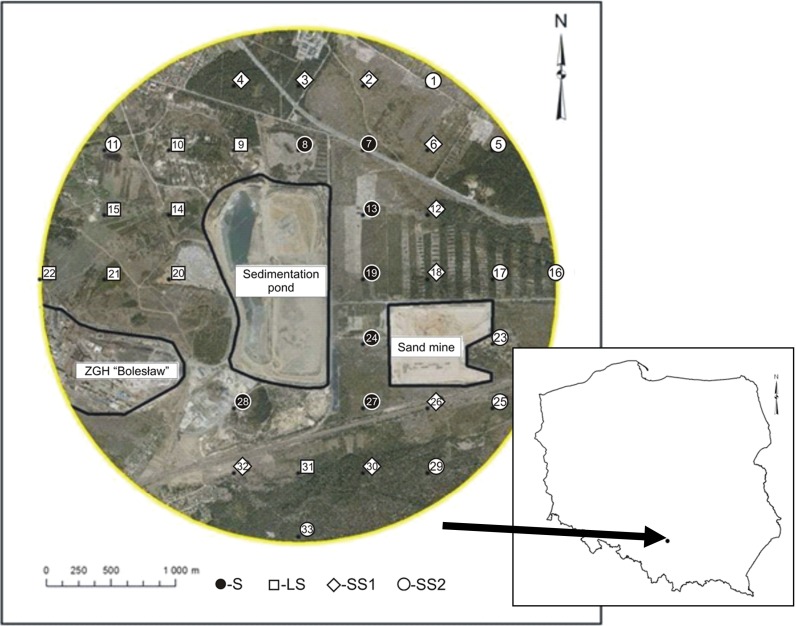
Location of the sampling points

The sampling points were divided into four groups, i.e. surfaces on the sandy substrate (sand is dominant fraction >90 %) approximately 500 m from the sedimentation pond (SS1-9 samples), surfaces on the sandy substrate situated approximately 1500 m from the sedimentation pond (SS2-9 samples), surfaces of the loam substrate (LS-8 samples; content 40 % silt and clay) and areas under the direct influence of sediment (S-7 samples) (Fig. 1). By comparison, control samples from areas that were not exposed to industrial pollution from sedimentation pond of lead and zinc were taken (CS-5 samples, control for sandy substrates; CL-5 samples, control for loam substrates). It was an unpolluted area with pine forest stands (50 km from the study area). Soils on the sandy substrates with control are podzols, and soils on the loamy substrates with controls are leptosols.

### Chemical Analysis

The soil samples that were obtained in the field were dried and then passed through a sieve with a mesh diameter of 2.0 mm. In each sample, chemical properties were determined, in two repetitions. The samples were measured for pH in H_2_O and KCl by the potentiometric method. The soil texture was determined by sieve method with the aerometric method according to the Polish norms: PN-R-04032 and PN-R 04033 (1998) (sand diameter was 2–0.05 mm, silt ø was 0.05–0.002 mm, clay ø <0.002 mm). The content of soil total carbon (C) was measured by elemental analyser, LECO CNS 2000. The content of trace elements (Zn, Pb and Cd) was determined by the emission spectrometry technique of inductively coupled plasma ICP-AES after prior mineralization in the mixture of concentrated nitric acid and perchloric at the ratio of 2:1 (Carter and Gregorich 2006).

### Enzyme Analysis

For the determination of enzymatic activity fresh samples of natural moisture (actual soil moisture content in percent of weight; it was comparable 8–14.5 %) were sieved through a sieve (ø 2 mm) and stored at 4 °C before the analysis. In the soil samples, the activity of dehydrogenase (EC 1.1.1.1) and urease (EC 3.5.1.5) were determined in three repetitions. The dehydrogenase activity (DH) was determined by the reduction of 2,3,5 triphenylotetrazolium chloride (TTC) to triphenyl formazan (TPF) using Lenhard’s method according to the Casida procedure (Alef and Nannipieri, 1995). Briefly, 6 g of soil was incubated with 1 ml of 3 % TTC for 24 h at 37 °C. The TPF was extracted with ethyl alcohol (95 %) that was contaminated with methanol. The TPF was measured spectrophotometrically (485 nm).

Urease activity (UR) was determined according to Tabatabai and Bremner (1972) with water urea solution as a substrate. This activity was determined by the NH_4_^+^ that was released after 2 h at 37 °C. The concentration of NH_4_^+^ was measured at 410 nm by the colorimetric method (Alef and Nannipieri, 1995).

### Community-Level Physiological Profiling (CLPP) Analysis

Biolog EcoPlates were used to evaluate the community-level physiological profile of different soils (Insam, 1997; Insam and Goberna, 2004). Briefly, 1 g of soil was shaken in 99 ml of distilled sterile water for 20 min at 20 °C and then incubated at 4 °C for 30 min (Pohland and Owen, 2009). Next, 150 μl of each sample was inoculated into each well of the Biolog EcoPlates and incubated at 25 °C. The rate of utilization was recorded with a plate reader at 590 nm every 24 h for 120 h. Based on overall dataset reading, mean values were calculated (*n* = 15).

The microbial response, regarded as overall respiration, in each microplate was expressed as the average well colour development (AWCD) (Garland and Mills, 1991; Gomez et al., 2004). To evaluate microbial functional diversity, richness (R) values were calculated as the number of oxidized C substrates using an optical density (OD) of 0.25 as the threshold for a positive response (Garland, 1997).

### Statistical Analysis

Maps of individual concentration distribution of heavy metals in the soil horizon were made (program ESRI ArcGIS). For this purpose, the IDW (inverse distance weighted) interpolation method was used (Isaaks and Mohan Srivastava, 1989).

To reduce the number of variables in the statistical dataset and to visualize the multivariate dataset as a set of coordinates in a high-dimensional data space, principal component analysis (PCA) method was used. PCA method was also used to interpret other factors, depending on the type of dataset. In PCA analysis, the chemical properties, enzymes activity of soil and distance to the source of contamination were used. Spearman’s correlation coefficients between enzyme activities and soil characteristics were also calculated. The multiple regression method was used to develop models describing the relationship between the estimated values of enzyme activity and soil characteristics. The differences between the mean values in the soil groups were evaluated with Tukey’s test (*P* < 0.05). All of the statistical analyses were performed with Statistica 9.0 software (2010).

The average well colour development and richness indices were analysed with ANOVA. Mean comparisons between the sampling point groups were performed using Tukey’s mean separation test at *P* < 0.05. To depict the functionality similarities between the microbial communities inhabiting each sampling point, UPGAMA clustering algorithm was used following Weber and Legge (2009).

## Results

### Chemical Characteristics of the Studied Soils

**Fig. 2:**
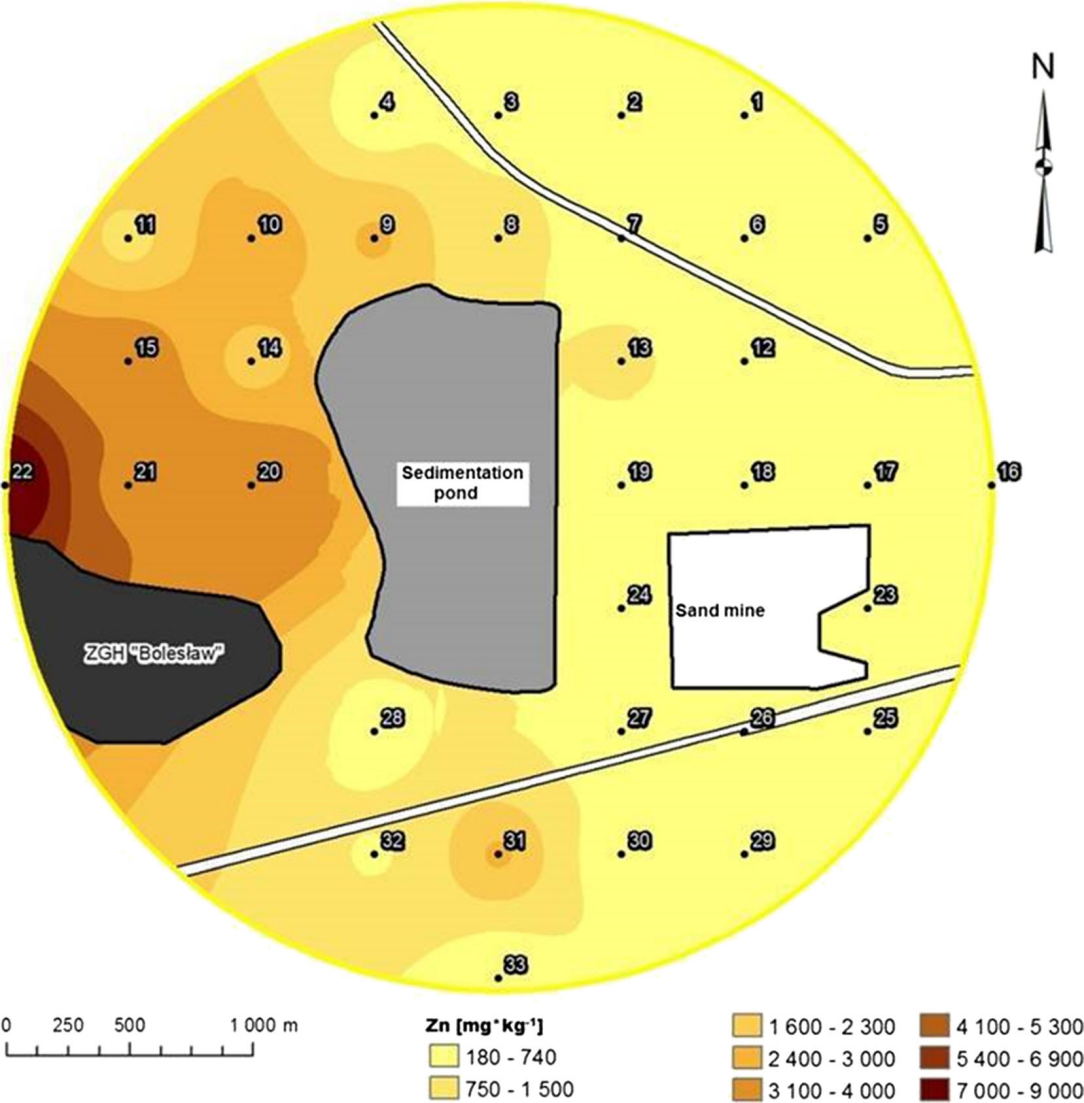
Distribution of the concentrations of zinc (Zn) in the topsoil in the study area

**Fig. 3:**
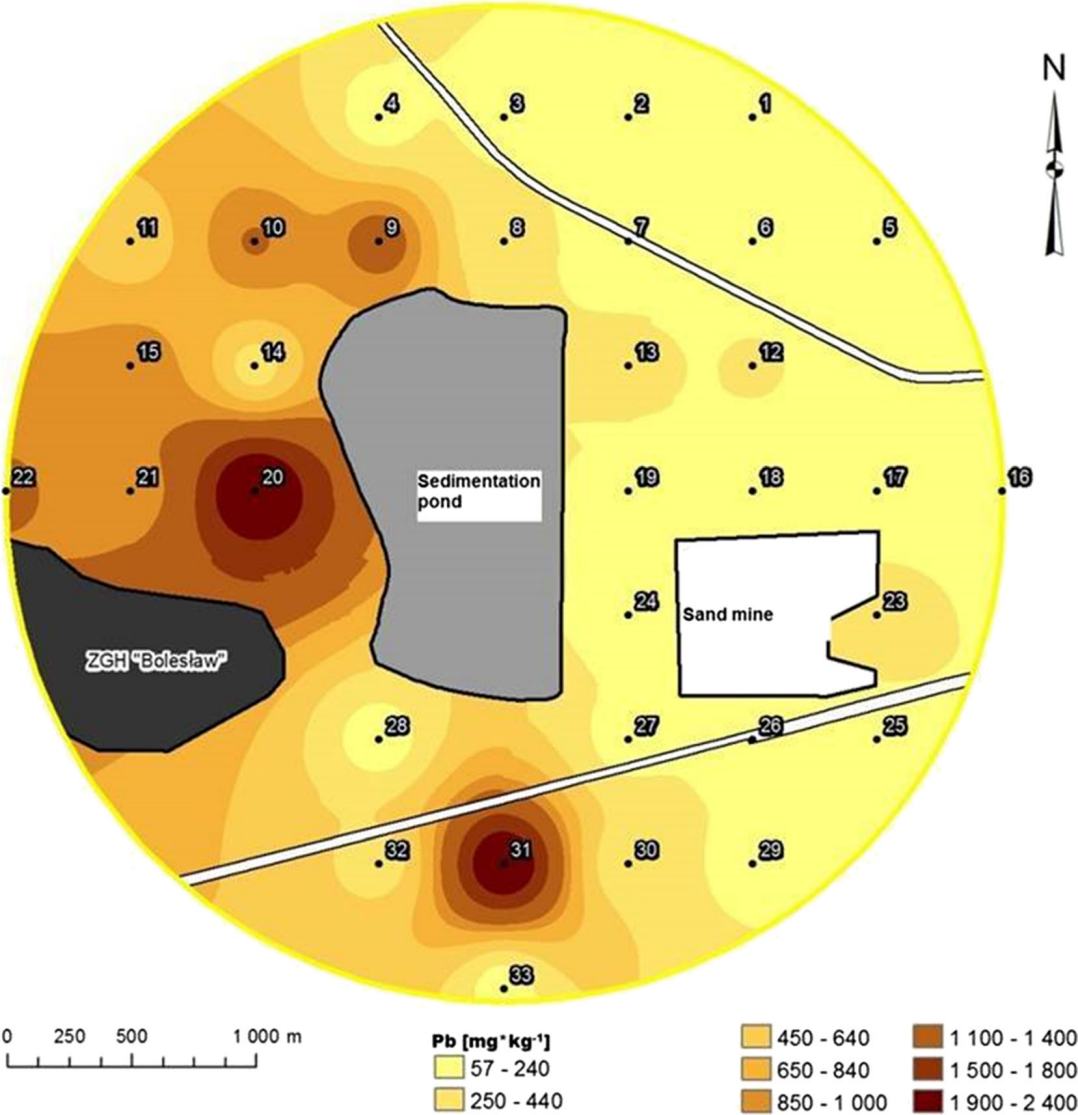
Distribution of the concentrations of lead (Pb) in the topsoil in the study area

The soils from the LS group were characterized as having the highest organic C content (Table 1). The soils from the SS2 group contained lower amounts of Zn and Pb than the soils from SS1 group and the soil area under the direct influence of sediment and soil from the loamy substrate (Table 1). Maps of metal concentration distributions in the soil illustrate the essential difference between the amount of metal that was deposited on the western side of the sedimentation pond on soils with high clay and silt fraction content and their quantities on the eastern side, where the substrates were light to very light (Figs. 2 and 3).

**Table 1:** The characteristics of soil taken from different group of substrates (SS1, SS2, LS, S)

	DH (μM TPF·kg^−1^ soil·h^−1^)	UR (mM N-NH_4_·kg^−1^ soil·h^−1^)	pH in H_2_O	pH in KCl	Organic C (%)	Sand	Silt	Clay	Zn	Pb	Cd
						(%)			(mg · kg^−1^ soil)		
SS1	^a^0.10 ± 0.14	^a^0.80 ± 0.04	^c^7.47 ± 0.69	^c^7.35 ± 0.89	^a^0.54 ± 0.44	^a^95 ± 1.8	^a^3 ± 1.5	^a^2 ± 0.7	^b^555.1 ± 145.8	^b^199.2 ± 91.8	^ab^3.3 ± 2.1
SS2	^a^0.38 ± 0.32	^a^1.16 ± 0.11	^b^6.24 ± 0.77	^b^5.64 ± 0.96	^a^0.52 ± 0.51	^a^95 ± 2.2	^a^1 ± 1.2	^b^4 ± 1.1	^b^220.8 ± 346.3	^b^107.1 ± 146.9	^a^1.5 ± 1.8
S	^a^0.27 ± 0.35	^a^1.23 ± 0.22	^b^6.80 ± 0.33	^b^6.48 ± 0.43	^a^1.99 ± 0.57	^a^91 ± 1.7	^a^5 ± 1.3	^b^4 ± 1.1	^b^960.5 ± 265.2	^b^278.6 ± 93.2	^b^9.7 ± 3.9
CS	^b^4.55 ± 2.08	^b^3.34 ± 1.96	^a^4.48 ± 0.20	^a^3.45 ± 0.15	^a^0.93 ± 0.57	^a^84 ± 1.2	^a^15 ± 1.8	^a^1 ± 0.6	^a^5.9 ± 3.8	^a^14.5 ± 7.8	^a^0.1 ± 0.1
LS	^a^11.17 ± 11.43	^a^2.59 ± 0.08	^a^7.63 ± 0.75	^a^7.40 ± 0.93	^a^4.38 ± 3.86	^a^60 ± 15.9	^a^31 ± 13.4	^a^9 ± 3.1	^b^3164.2 ± 2253.2	^b^1202.8 ± 711.3	^b^15.7 ± 10.3
CL	^b^39.31 ± 4.0	^b^6.80 ± 1.43	^a^6.59 ± 0.19	^a^6.34 ± 0.16	^a^5.38 ± 0.43	^a^54 ± 3.9	^a^33 ± 2.3	^a^12 ± 2.6	^a^44.84 ± 8.79	^a^15.71 ± 10.01	^a^0.36 ± 0.08

mean ± standard deviation; *DH* dehydrogenase activity, *UR* urease activity, *SS1* surfaces on the sandy substrate—approximately 500 m from the sedimentation pond, *SS2* surfaces on the sandy substrate situated approximately 1500 m from the sedimentation pond; *LS* surfaces of the loam substrate, *S* areas under the direct influence of sediment, *CS* control for surfaces on the sandy substrate, *CL* control for surfaces on the loam substrate; *different small letters* in the upper index of the mean values mean significant differences

**Fig. 4:**
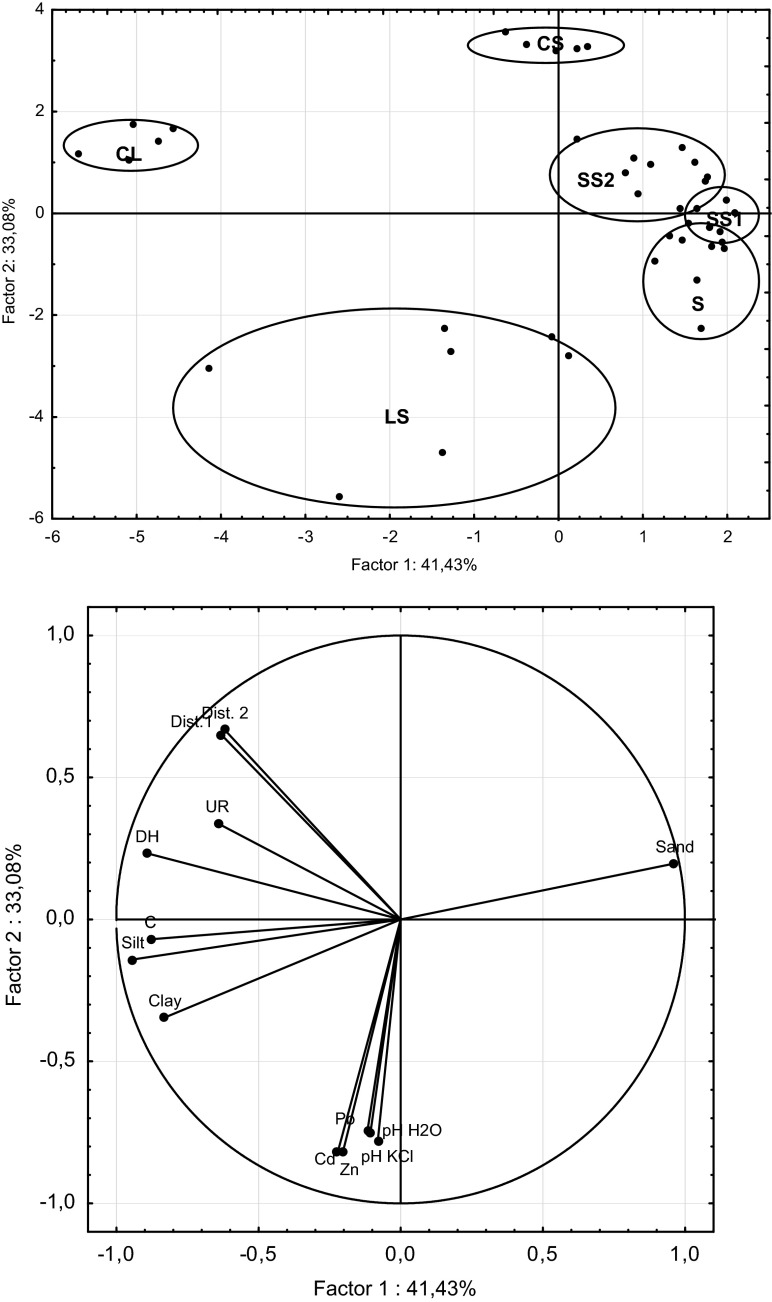
Factorial plan and projection of variables in the soil properties on the factor-plane 1 × 2

PCA analysis showed strong variability in the sampling points in terms of particle size. The results that are summarized in Fig. 4 indicate the separation of sampling points with fine grain size (LS). The content of the silt and clay fractions determines the quantity of the accumulated heavy metals. Additionally, the control sampling points for sandy and loam substrates were separated. Studies have shown that samples with fine fractions (ø <0.002 mm) contained metals. Sandy soils were characterized by lower concentrations of metals. The results that are summarized in Fig. 4 show that the carbon content was correlated with the content of fine fractions.

### Enzyme Activity of the Studied Soils

The highest enzyme activity was found in the soils from LS group, and the lowest activity was found in the soils from the SS1 group (Table 1). The greatest reduction in enzyme activity was noted in sandy soils with contents of Zn >220 mg · kg^−1^ and Pb >100 mg · kg^−1^. In the soils with high clay and silt fraction content, also the reduction in microbial activity was observed (Table 1). Enzyme activity (particularly dehydrogenase) correlated with the carbon content (Table 2, Fig. 4). A negative correlation of soil pH with the distance from the sedimentation pond and the activity of soil enzyme was found (Table 2, Fig. 4). Urease and dehydrogenase activities were associated with the distance from the sedimentation pond (Dist. 1) (Table 2). The activity of the studied soil increased with the distance from the sedimentation pond (Table 2, Fig. 4). Multiple regression models explained from 48 to 78 % of the variance in the enzyme activity (Table 3). Dehydrogenase activity of soil correlates negatively with Pb content and positively with organic C content. For urease activity, the most important related variables were C content, pH and Pb content (Table 3).

**Table 2:** Correlations between enzyme activities and soil characteristics

	Zn	Pb	Cd	pH_H20_	pHKCl	C	Sand	Silt	Clay	Dist. 1	Dist. 2
DH	−0.33*	−0.27	−0.19	−0.28	−0.30	0.55*	−0.63*	0.56*	0.51*	0.53*	0.30
UR	−0.44*	−0.39*	−0.38*	−0.40*	−0.41*	0.34*	−0.47*	0.42*	0.31*	0.63*	0.47*

*DH* dehydrogenase activity, *UR* urease activity

*P < 0.05

**Table 3:** Multiple regression analysis for enzymes activity based on soil characteristic. *R* ^2^ describes the percentage of explained variance, *β* is the regression coefficient for given equation parameter and *p* is the significance level for the equation parameter

Enzyme activity	*R* ^2^	Equation parameter	*β*	*p*
DH	78 %	Pb	−0.0082	0.000024
		Organic C	6.1631	0.000010
UR	48 %	Organic C	0.8977	0.000013
		pH_H2O_	−0.3677	0.007790
		Pb	−0.0024	0.004631

*DH* dehydrogenase activity, *UR* urease activity

### Community-Level Physiological Profiling (CLPP) Analysis

**Fig. 5:**
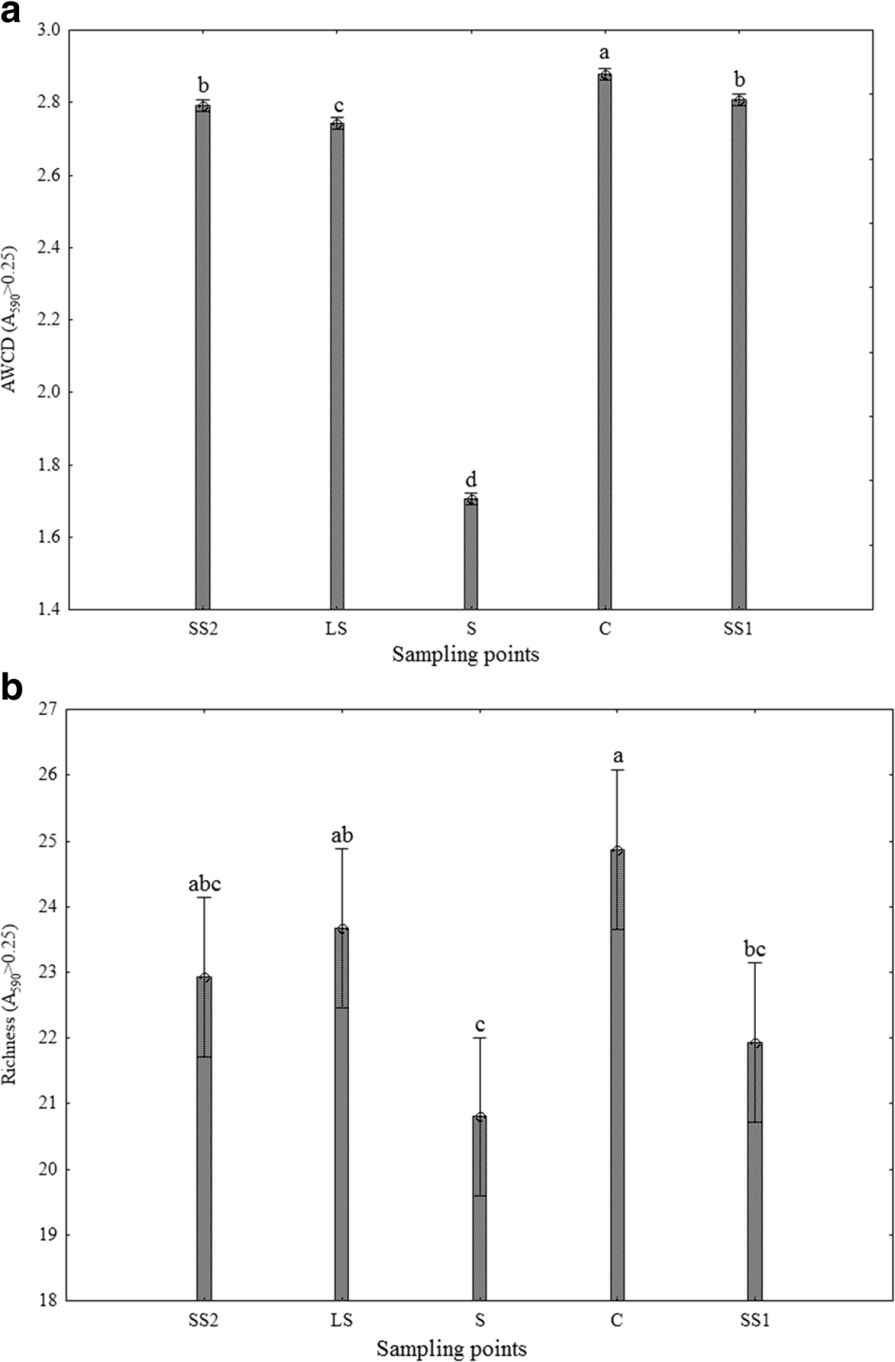
Microbial functional parameters in the topsoil of the study area (a) average well colour development (AWCD) and (b) richness (R) within Biolog EcoPlate. *Vertical bars* denote 0.95 confidence intervals (*n* = 15). Different letters indicate significant differences (*P* < 0.05)

The results that are shown in Fig. 5a, b demonstrate that the indicators AWCD and R that were used in the evaluation of soil microbial activity and functional diversity, respectively, were lowest in the soil under the direct influence of sediment (S). The highest values of AWCD and R indices were observed in the control soil collected from areas that were not exposed to industrial pollution. Significant inhibition of total metabolic activity, as expressed by AWCD index, observed in the areas under the direct influence of sediment compared with other treatments, could indicate lower maintenance energy due to the microbial community stress caused by heavy metals (Zn, Pb) contamination. Three times higher values of AWCD in surfaces on the sandy substrate located in the distance of 500 m (SS1) and 1500 m (SS2) from the sedimentation pond and surfaces of the loam substrate (LS), compared with the S sampling point may indicate a greater adaptive capacity and faster use of carbon substrates by soil microbial communities. However, the demonstrated results for the abovementioned sampling points were still significantly lower than in the reference control soil. The results of richness indicate that the functional response of microbial populations was similar in samples C, SS2 and LS while they differed significantly from S and SS1.

**Fig. 6:**
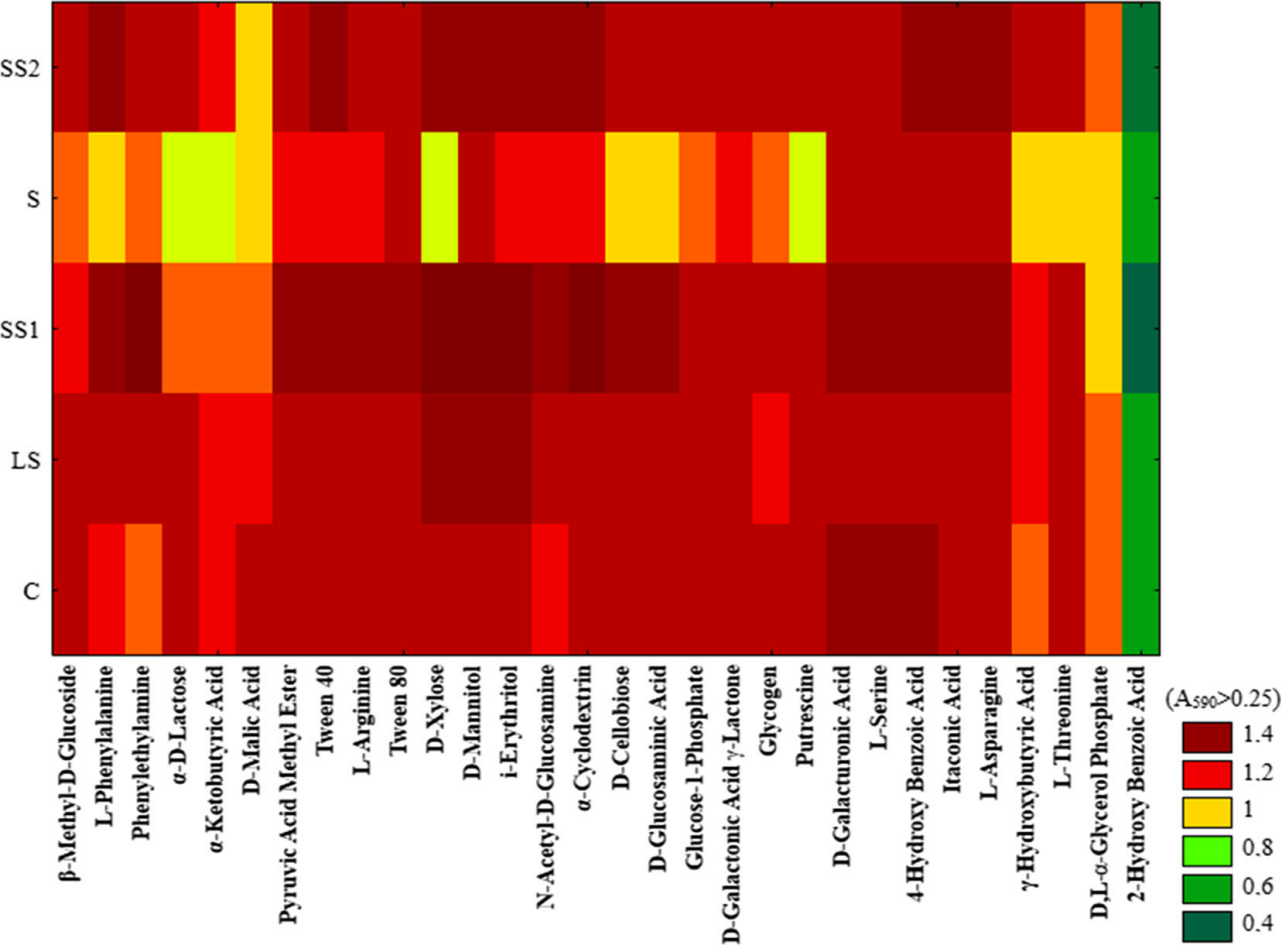
Results of cluster analysis of different soils based on carbon source substrate utilization in EcoPlate

The study showed that most of the analysed carbon substrates were used extensively in many of the soil samples. Ecological meaning of results presented at Fig. 6 is connected not only with the quantity evaluation of microbial functional response but also with the quality differentiation of the most physiologically active communities based on catabolic fingerprint. Significantly lower levels of substrate utilization were found in the soil under the direct influence of sludge than in the control and other soil groups (SS1, SS2 and LS). Reduced metabolic activity in the S sample was established for the following substrates: l-phenylalanine, α-d-lactose, α-ketobutyric acid, d-malic acid, d-xylose, d-cellobiose, d-glucosaminic acid, putrescine, hydroxybutyric acid, l-threonine and dl-α-glycerol phosphate. 2-Hydroxybenzoic acid was used in all of the samples at very low levels. Furthermore, a lower activity of d-malic acid was found in the SS2 sample, while a lower activity of dl-α-glycerol phosphate was found in the SS1 sample. The most intense metabolism in many of the analysed substrates was found in the soil that was collected from a sandy substrate closest to the sedimentation pond as well as in soil from the sandy substrate farthest from the sedimentation pond. α-Cyclodextrin, phenylethylamine, d-xylose, d-mannitol and i-erythritol were the most utilized substrates (Fig. 6).

**Fig. 7:**
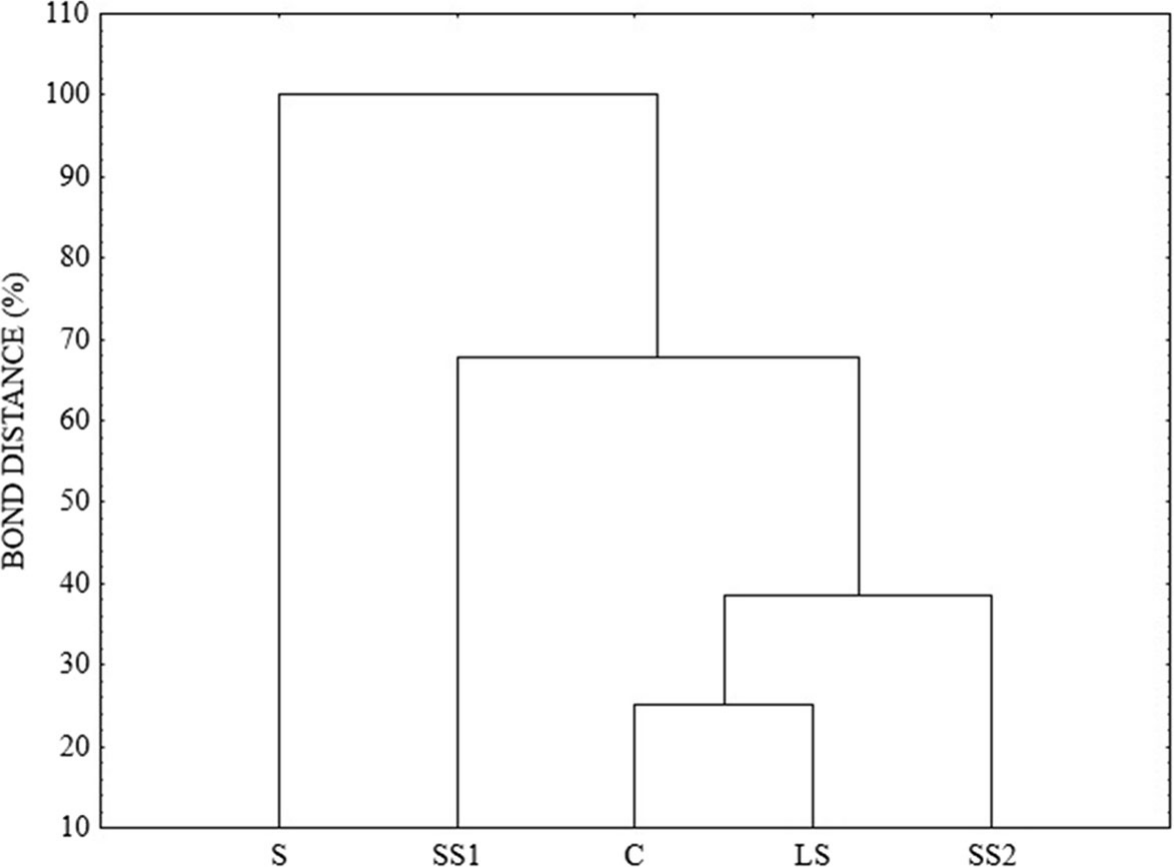
Results of cluster analysis for different soils based on metabolic profiles

Based on the cluster analysis, the examined soil can be divided into two groups (Fig. 7). The soil from the zone with direct sediment impact was included in the first group. In the second group, we distinguished two subgroups, one of which is the soil from the sandy substrate near the sedimentation pond. These results indicate that under the influence of sedimentation pond microorganisms communities can change the metabolic profile of soil and can cause a reduction of functional diversity. These changes disturb the ecological balance of the forest ecosystem.

## Discussion

### Relationships Between Soil Chemical Properties and Enzyme Activity

Very high concentrations of Zn, Cd and Pb were observed in the soils from the loamy substrate and soils under the direct influence of sediment. The concentrations of these elements exceeded the intervention level of the Dutch list (1996). Under this regulation, the maximum allowable concentrations of Zn, Cd and Pb in the soils of industrial areas should not exceed 720, 12 and 530 mg · kg^−1^, respectively.

The analysis of metal concentration at the surface horizon of soil showed differences in the amount of absorbed elements, depending on the particle size and carbon content in the soil. Heavy metals and enzymatic activities are associated with carbon content in the soil and the participation of sand, silt and clay (Kandeler and Eder, 1993). Renella et al. (2003) reported that Cd solubility was greater in soils with a coarser texture and lower organic matter content. According to Renella et al. (2007), the stimulatory effects of some low-molecular-weight organic compounds on the microbial activity and hydrolase activity depend on the type of soil, especially the soil texture and pH.

In the study, substrates with more fine fractions and those that were richer in carbon were characterized by a higher amount of accumulated metals and stronger contamination. A correlation between the activity of dehydrogenases and carbon content was reported. It can be assumed that organic matter is an energy source for microorganisms and the substance-complexing heavy metals. In addition, dehydrogenase activity was associated with the type of substrate and the percentage share of each fraction in the soil. Soils containing more organic carbon and clay colloids create better conditions for microorganisms, and their microbiological and biochemical activities are greater. The results of this study indicate that heavy metal pollution in connection with soil properties especially C content, pH and texture affects the soil microbiological parameters. Variability of soil properties such as pH, C content and texture significantly influence the mobility and potential availability of heavy metals. Soils with higher content of fine fraction and carbon have absorbed more heavy metals but their mobility and availability in soil is lower. In effect, soil microbial activity is not significantly limited. Light sandy soils have a low capability of heavy metal absorption. The study reported very low activities of dehydrogenase and urease in the soils in the poorest pine forests (Błońska, 2011). Heavy metals can reduce enzyme activity by interacting with the enzyme-substrate complex, denaturing enzyme protein or interacting with the protein-active groups (Nannipieri, 1994). Heavy metals can also affect enzyme synthesis in microbial cells. In our study, a decrease in urease activity was noted. The inhibitory influence of heavy metal pollution (Cu, Pb, Zn and Cd) on urease activity was reported by Zheng et al. (1999). Moreno et al. (2001) suggested that Cd inhibits the activity of extracellular ureases as stabilized by soil colloids.

Enzymes positively correlate with the distance from the sedimentation pond—the source of metals. According to Šmejkalova et al. (2003), a decreasing distance from the source of contamination caused a decrease in the enzymatic activities in the soil. Wang et al. (2007) observed a good correlation between enzyme activity and the distance from the copper-zinc smelter. These authors noted a significant decrease in the enzyme activity in the soils 200 m from the smelter. A negative correlation between the soil pH and the distance from the sedimentation pond and soil enzyme activity resulted from the nature of dust-borne contaminants. In this area, Krzaklewski et al. (2004) observed that metals are carried by the wind with dust, accumulating in the vicinity of ponds at the surface horizons. The farther from the sedimentation pond, the less alkaline the dust fallout, which results in a lower soil pH and higher acidity. The reaction, in this case, is not a factor that enhances the activity of microorganisms because the inhibitory impact of toxic components is stronger.

Our results demonstrate the significant inhibition of enzymatic activity that is caused by high levels of metals in connection with soil properties (carbon content, texture and pH). Enzyme activity could be a good indicator of soil quality because it is sensitive and reflects biological situation in the soil and is strongly correlated with important soil characteristics, such as organic matter and soil texture.

### Relationships Between the Community-Level Physiological Profiling (CLPP) Analysis and the Soil Chemical Characteristics

In this study, the analysis of the Biolog® data for the sole carbon source utilization pattern demonstrated that heavy metal pollution had a significant impact on the microbial functional diversity and overall metabolic activity, expressed as richness index and AWCD, respectively. Biolog®-derived parameters simultaneously depended on various soil properties. The soils with a higher concentration of fine fraction, despite having the highest concentration of metals, were characterized by high rates of R and thus high enzyme activity. This effect was especially noticed in the soil collected from loam substrate sampling point, where despite the highest content of Zn and Pb, high fine fraction content probably mitigated their negative influence on microbial functionality, which was noted as high values of such parameters as AWCD and R.

The AWCD reflects the average microbial community metabolic response as the ability to utilize the substrates that are located on the EcoPlate®. The reduction of microbial activity, as shown by AWCD for heavy metal-treated soils, can be indirectly explained as the consequence of negative effects of heavy metals, probably resulting in the reduction of the numbers and/or species diversity of the biota (Akmal et al., 2005). The reduction of functional diversity in the zone with sediment impact and the creation of a separate group of soil microorganisms in that area indicate the disturbance of homeostasis in the ecosystem as a result of the impact of waste and accumulated Zn, Pb and Cd. These results confirm that the Biolog plate technique can be used to evaluate the impact of stressing factors, such as heavy metals, as a result of bad management. According to Gomez et al. (2004, 2006) the estimation of microbial functional diversity could be an approach to detect modifications due to soil management.

## Conclusions

The results indicate that heavy metal pollution and soil characteristics had a significant impact on the soil microbiological parameters. Heavy metal pollution significantly reduced soil dehydrogenase and urease activity. Concentration of metal depends on the soil characteristics, such as texture, carbon content and distance to the source of contamination. The increase in clay and organic carbon content causes less negative impact of Zn and Pb on microbial activity. The greatest reduction in enzyme activity was observed in light sandy soils with a Zn content >220 mg · kg^−1^ and Pb content >100 mg · kg^−1^. Soils with a higher concentration of fine fraction, despite having the highest concentrations of metals, were characterized by high rates of Biolog®-derived parameters and thus less reduction of enzyme activity. Decreasing distance from the source of contamination caused a decrease in the soil microbial activity. Enzymes positively correlate with the distance from the sedimentation pond – the source of metals.
